# An Analysis of the Influence of Surface Roughness and Clearance on the Dynamic Behavior of Deep Groove Ball Bearings Using Artificial Neural Networks

**DOI:** 10.3390/ma16093529

**Published:** 2023-05-04

**Authors:** Ivan Knežević, Milan Rackov, Željko Kanović, Anja Buljević, Aco Antić, Milan Tica, Aleksandar Živković

**Affiliations:** 1 Department of Mechanization and Design Engineering, Faculty of Technical Sciences, University of Novi Sad, 21000 Novi Sad, Serbia; ivanknezevic@uns.ac.rs; 2 Department of Computing and Control Engineering, Faculty of Technical Sciences, University of Novi Sad, 21000 Novi Sad, Serbia; kanovic@uns.ac.rs (Ž.K.); anjabuljevic@uns.ac.rs (A.B.); 3 Department of Production Engineering, Faculty of Technical Sciences, University of Novi Sad, 21000 Novi Sad, Serbia; antica@uns.ac.rs (A.A.); acoz@uns.ac.rs (A.Ž.); 4 Department of Mechanics and Construction, Faculty of Mechanical Engineering, University of Banja Luka, 78000 Banja Luka, Bosnia and Herzegovina; milan.tica@mf.unibl.org

**Keywords:** bearing, neural network, surface roughness, clearance, vibration

## Abstract

The deep groove ball bearing is one of the most important components of the rotary motion system and is the research subject in this paper. After factory assembly, new ball bearings need to pass quality control. The conventional approach relies on measuring the vibration amplitudes for each unit and sorting them into classes according to the vibration level. In this paper, based on experimental research, models are created to predict the vibration class and analyze the dynamic behavior of new ball bearings. The models are based on artificial neural networks. A feedforward multilayer perceptron (MLP) was applied, and a backpropagation learning algorithm was used. A specific method of training groups of artificial neural networks was applied, where each network provided an answer to the input within the group, and the final answer was the mean value of the answers of all networks in the group. The models achieved a prediction accuracy of over 90%. The main aim of the research was to construct models that are able to predict the vibration class of a new ball bearing based on the geometric parameters of the bearing rings. The models are also applied to analyze the influence of surface roughness of the raceways and the internal radial clearance on bearing vibrations.

## 1. Introduction

Deep groove ball bearings are one of the most important components of rotary motion systems, and the vibrations generated by the bearing have a significant influence on the dynamic behavior of the system. When designing complex rotary motion systems, whose integral parts are deep groove ball bearings, it is very important to know the dependence of vibrations and the many factors that affect them. The vibration measurement method is prescribed by the ISO 15242-2 standard. New ball bearings need to pass quality control after assembly in the factory. The conventional approach involves measuring the vibration amplitudes for each unit and sorting them into classes according to the vibration level. Mathematical models are used to describe the dynamic and static behavior of bearings [1,2,3,4]. One of the first studies of the influence of the surface roughness of raceways on bearing vibrations was conducted by Kanai et al. [5]. They developed a method for determining the surface roughness of a bearing’s raceway based on an analysis of the bearing’s vibration signal. Yunlong et al. [6] developed a dynamic model to determine the dynamic behavior and movement of the rolling elements of the bearing based on the surface roughness at the point contact of the ball and the raceway. Zmarzly [7] investigated the effect of surface roughness and raceway waviness on bearing vibrations. Using linear regression, the author approximated the dependence of bearing vibration amplitudes on the surface roughness and raceway waviness.

Artificial neural networks have been used for a long time for the vibration analysis of deep groove ball bearings. Their basic application is to determine the cause of bearing faults. Today, there are solutions that are practically applied for bearing fault diagnosis. A review on the application of convolutional neural networks in bearing fault diagnosis is provided in [8], and an overview of applied artificial intelligence techniques for bearing analysis and fault diagnosis was presented by Liu et al. [9]. The application of artificial neural networks for predicting vibration amplitudes of deep groove ball bearings based on technological parameters and lubrication is presented in [10]. Artificial neural networks have been successfully applied to predict the size of the internal radial clearance in deep groove ball bearings, as presented in reference [11]. Different examples of the application of artificial intelligence to detect fault in bearings are presented in [12,13,14,15,16,17]. The authors in [18,19,20,21,22] use artificial neural networks to predict the remaining useful life of the bearing (RUL).

In [23], the authors apply artificial deep learning neural networks to predict the surface roughness in metal processing. The signals were transformed using FFT (fast Fourier transformation) into a form suitable for training artificial neural networks. Lin et al. [24] analyzed metal processing parameters using artificial neural network models to predict the surface roughness of the processed surface.

Predictive maintenance is an important strategy in rotating machinery to ensure high reliability of the system. Today, there are a number of different methods that help to improve the reliability of rotary systems. The most used is analysis of measured vibration signals with different artificial intelligence tools. In [25], the authors reviewed methods used in prediction of the maintenance requirements of gearboxes, bearings and generators of wind turbines. Lalik and Watorek [26] presented a neural control algorithm for defect detection of wind turbine rotary systems. They used artificial intelligence to determine the technical condition and predict possible failure in the system.

The main aim of this research was to construct models able to predict the vibration class of a new ball bearing based on the geometric parameters of the bearing rings. The models were also applied to analyze the influence of surface roughness of the raceways and internal radial clearance on bearing vibrations. Based on experimentally obtained data, groups of artificial neural networks were trained and were used to predict the vibration amplitude of deep groove ball bearings depending on the geometric characteristics of the raceway and internal radial clearance. The axial load was constant for this experimental research and was not considered as an influential factor. In the available literature, no such approach to the investigation of the dynamic behavior of ball bearings was found. To date, this is the first study to utilize artificial neural networks to predict the vibration class of new ball bearings based on the geometric parameters of the bearing rings and to investigate the dependence of surface roughness and vibrations, as well as clearances and vibrations in the bearing.

## 2. Materials and Methods

**Figure 1:**
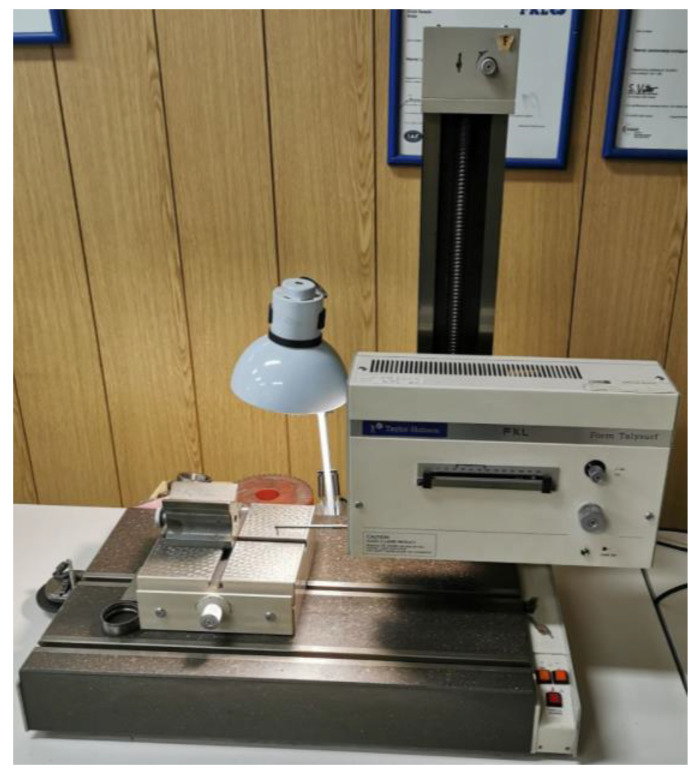
Taylor–Hobson Form Talysurf 112/1849 Device.

The test sample consisted of 30 deep groove ball bearings marked 6006. The experiment involved measuring the geometric deviations and surface roughness of the raceway of the inner and outer rings, the internal radial clearance, as well as the vibration amplitudes of the ball bearings. Geometric deviations were measured using the Taylor-Hobson Form Talysurf 112/1849 device as shown in Figure 1. The statistical features of the geometric deviations and internal radial clearance are presented in Table 1. These include the internal radial clearance (G_r_), raceway radius ratio (R_i_/R_e_), surface roughness of the raceway of the outer ring (R_ae_), raceway waviness of the outer ring (W_te max_), deviation from the circularity of the raceway of the outer ring (W_te_), surface roughness of the raceway of the inner ring (R_ai_), raceway waviness of the inner ring (W_ti max_), and deviation from the circularity of the raceway of the inner ring (W_ti_).

The geometric deviation parameters of the raceways for the inner and outer rings are independent since each ring is made independently of the others. When selecting the inner and outer rings for mounting the bearing, it is possible that the geometrical parameters are completely different. To eliminate the influence of different parameters, equivalent parameters were introduced, and their value was calculated using the following expressions:

The equivalent surface roughness of the raceway (R_a ekv_):

(1) $$R_{a\;\mathrm{ekv}}=\frac{R_{ae}\cdot R_{ai}}{R_{ae}+R_{ai}},$$

The equivalent amplitude of raceway waviness (W_t max ekv_):

(2) $$W_{t\;\max\;\mathrm{ekv}}=\frac{W_{te\max}\cdot W_{ti\max}}{W_{te\max}+W_{ti\max}},$$

The equivalent amplitude of deviation from the circularity of raceway (W_t ekv_):

(3) $$W_{t\;\mathrm{ekv}}=\frac{W_{te}\cdot W_{ti}}{W_{te}+W_{ti}}.$$

Table 2 shows the main statistical characteristics of equivalent bearing parameters.

**Figure 2:**
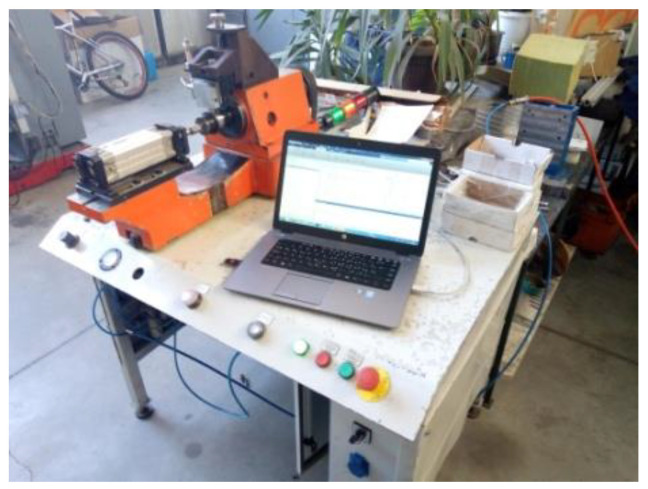
Experimental device for measuring ball bearing vibrations.

**Figure 3:**
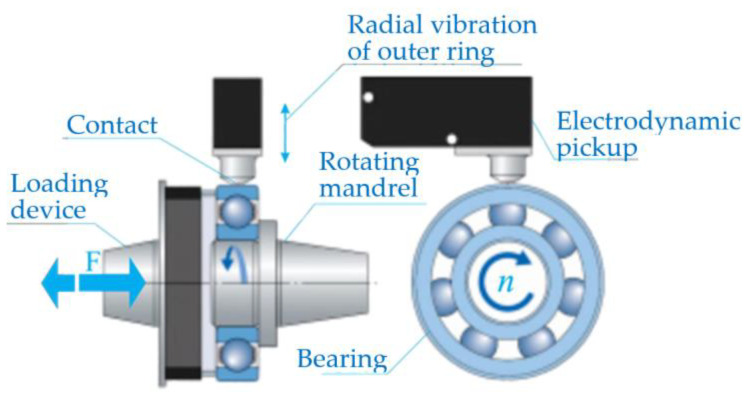
Schematic view of the vibrations measuring principle using the electrodynamic pickup.

The measurement of vibration velocity amplitudes was carried out using a device for measuring and analyzing bearing vibrations. Figure 2 shows the experimental device consisting of a test table on which there is a spindle mounted with hydrodynamic bearings, a pneumatic cylinder for axial loading of the bearing, a drive motor and a control cabinet (control unit). The bearing is placed over the inner ring on the measuring mandrel, which is connected to the spindle by means of a cone and a threaded connection. During the measurement, the spindle rotates at a constant number of revolutions (n = 1800 RPM) with a permissible deviation of +1% and −2%, according to ISO 15242-1. The outer ring is stationary and loaded with an axial force via a pneumatic cylinder, as standard ISO 15242-21 recommends. The basic element in the vibration measurement chain is the electrodynamics pickup (Figure 3) that generates a voltage at its output, and whose amplitude and frequency is proportional to the velocity of the vibrations generated by the ball bearing. The electrodynamic pickup is connected to a computer by a USB cable, which controls the measurement process and stores the measured signals. The measurement takes 5 s from the moment the bearing reaches a constant rotation velocity. Since the amplitude of the signal obtained by the electrodynamic pickup used is small, the signal is amplified by an amplifier for digital processing and display.

**Figure 4:**
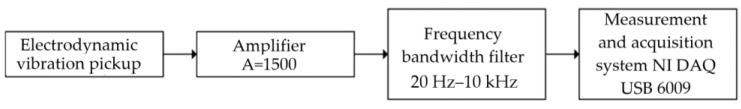
Block diagram of the measurement and control system.

A block diagram illustrating the measurement and control system for the vibration testing of ball bearings is shown in Figure 4. The elements responsible for analogue signal processing are the amplifier and the bandwidth filter. The amplifier has the task of amplifying the signal level from the electrodynamic velocity sensor to a level suitable for digital processing and display. An amplifier with a gain of 1500 was used, providing sufficient signal amplitude for digital processing. The frequency band of the signal that is of interest for vibration testing of ball bearings is from 50 Hz to 10 kHz. The filter functions to limit the spectrum of the signal received from the amplifier to the mentioned band. The designed filter introduces a relatively small attenuation of wave oscillations in one or more frequency bands and a relatively large attenuation for oscillations of other frequencies (below 50 Hz and above 10 kHz) according to the ISO 15242-1 standard. The bandwidth of the filter is also defined based on the mentioned standard. Signal digitization is performed using the NI DAQ USB-6009 measurement acquisition system. The sampling frequency is 48 kHz, while the resolution of the internal A/D converter is 13 bits. In this way, the quality preparation of the signal obtained using the electrodynamic velocity sensor and its digitization for further computer processing is ensured.

The signal recorded in the time domain is transformed into the frequency domain for further analysis using fast Fourier transformation. The standard requires the analysis of vibrations in three characteristic frequency bands, namely, low frequencies (50 ÷ 300 Hz), medium frequencies (300 ÷ 1800 Hz), and high frequencies (1800 ÷ 10000 Hz).

### 2.1. Analysis of Measured Data

**Figure 5:**
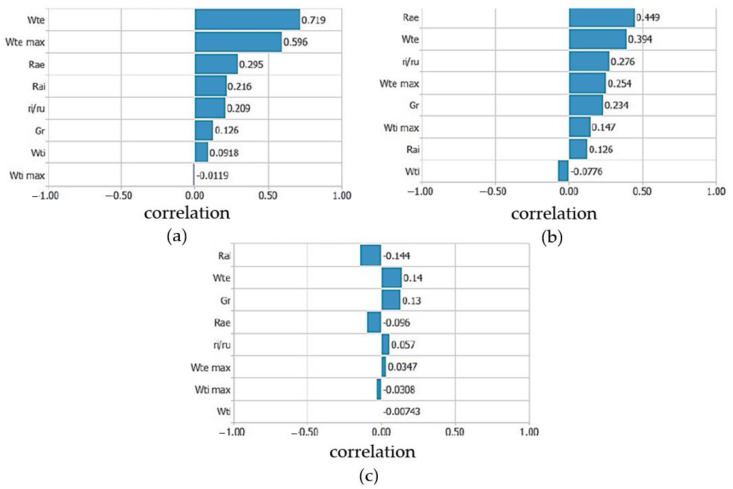
Linear correlation coefficients of bearing parameters and frequency bands (**a**) low-frequency band; (**b**) medium-frequency band; (**c**) high-frequency band.

The measurements of the geometric characteristics of the bearing raceway and the amplitude of the vibration velocity were analyzed to find mutual dependencies. The analysis was performed by calculating the coefficients of linear correlation between the characteristic parameters of the bearing and the amplitude of the vibration velocity in the frequency bands. In this analysis, the input parameters were the geometric characteristics of the bearing raceway, and the output velocities of vibrations in characteristic areas. The analyzed cases were those when the geometric characteristics of the bearing raceway were observed for each ring separately and when equivalent parameters were used. Figure 5 shows the values of the linear correlation coefficient of the characteristic bearing parameters where the technological parameters are observed for the outer and inner ring separately according to the characteristic frequency bands. The linear correlation coefficients of the bearing parameters for the low-frequency band are shown in Figure 5a. The highest correlation coefficient was found for the deviation from circularity of the outer ring, followed by waviness and surface roughness of the outer ring. The next most important influencing parameter was the surface roughness of the inner ring, followed by the ratio of the radius of the raceway, the radial clearance, the deviation from the circularity and the waviness of the inner ring.

Figure 5b shows the linear correlation coefficients of the bearing parameters for the medium-frequency band. The surface roughness of the outer ring has the highest influence, followed by the deviation from the circularity of the outer ring, the ratio of the radius of the raceway, the waviness of the outer ring, the radial clearance, and further parameters of the inner ring of the bearing. The correlation coefficients are lower compared with the correlation coefficients in the low-frequency band.

The linear correlation coefficients of the bearing parameters for the high-frequency band are shown in Figure 5c. The values of the coefficients are significantly lower compared with the low and medium frequency bands. The surface roughness of the inner ring, the deviation from the circularity of the outer ring and the radial clearance have the highest influence. Parameters with low influence are the surface roughness of the outer ring, the ratio of the radius of the raceway, the waviness of both rings and the deviation from the circularity of the inner ring.

**Figure 6:**
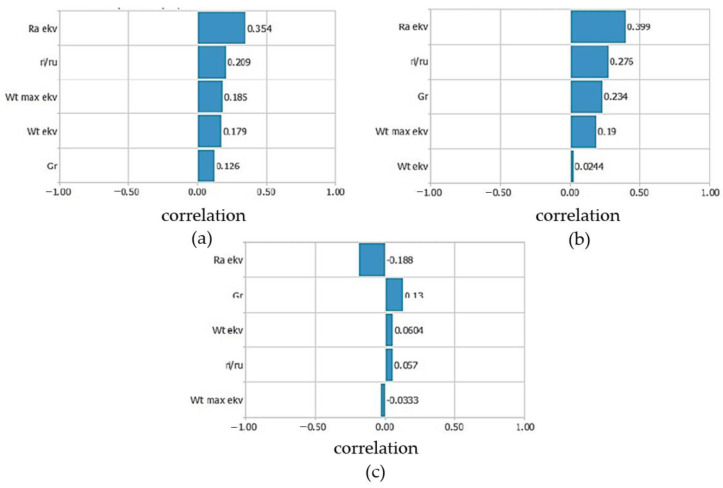
Linear correlation coefficients of bearing parameters and frequency bands (equivalent technological parameters): (**a**) low-frequency band; (**b**) medium-frequency band; and (**c**) high-frequency band.

Figure 6 shows the linear correlation coefficients of the characteristic bearing parameters and equivalent technological parameters in the three frequency bands. Figure 6a shows the linear correlation coefficients of the bearing parameters for the low-frequency band. The highest correlation coefficient was observed for the equivalent surface roughness, followed by the ratio of the radius of the raceways. Equivalent waviness and equivalent deviation from roundness were the next two influencing parameters. Radial clearance had the least influence in the low-frequency band.

Figure 6b shows the linear correlation coefficients of the bearing parameters for the medium-frequency band. The equivalent surface roughness has the highest influence, followed by the ratio of the radius of the raceways and the radial clearance. The less influential parameters were equivalent waviness and deviation from circularity.

The linear correlation coefficients of the bearing parameters for the high-frequency band are shown in Figure 6c. The values of the coefficients were also significantly lower compared with those for the low and medium-frequency bands. The equivalent surface roughness had the highest influence, which affects the reduction of the vibration velocity amplitudes. Radial clearance was the next most important influencing parameter. The parameters with lower influence were the equivalent deviation from circularity, the ratio of the radius of the raceways and the equivalent waviness.

### 2.2. Application of the Neural Network

Artificial neural networks are used in this paper to investigate the influence of the surface roughness of the raceways and the clearance in the bearing on the vibration velocity amplitudes. The paper applies a multilayer perceptron (MLP), where a feedforward signal and a learning algorithm with error backpropagation are used. The neural network has one hidden layer with d inputs, k neurons in the hidden layer, and one output, and the total ratio of inputs to outputs in this case is a function of $f:{\mathbb{R}}^{d}\to \mathbb{R}$ which maps the input vector $x\in {\mathbb{R}}^{d}$ into a scalar output using the following equation [27]:

(4) $$x\to f_{v,}w\left(x\right):=\sum_{l=1}^{k}v_{l}\phi \left(\left(w_{l},x\right)\right)$$

Vectors $w_{l}\in {\mathbb{R}}^{d}$ contain weighting coefficients between the inputs and l-th hidden node, and $v_{l}\in {\mathbb{R}}^{d}$ is the weight coefficient of the *l*-th hidden node and the output. Finally, ϕ:ℝ→ℝ denotes the activation function applied to each hidden node.

### 2.3. Organization of Data Set

**Figure 7:**
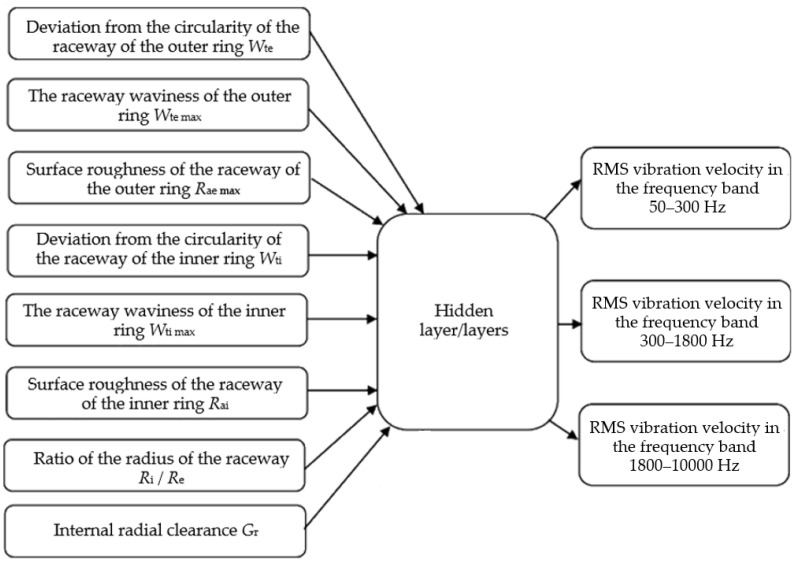
Structure of I/O parameters of the artificial neural network.

**Figure 8:**
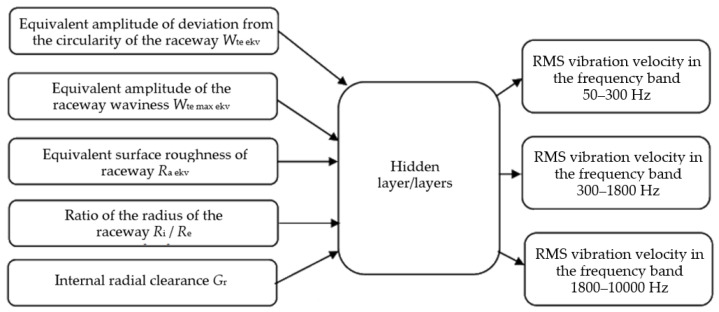
Structure of I/O parameters of the artificial neural network; input parameters are considered together (equivalent technological parameters).

The results of the experimental research are divided into groups of I/O parameters for the application of the artificial neural network. The input parameters are the geometric characteristics of the bearing and external load, and the output results are the vibration measurements of the bearing as shown in Figure 7. Figure 8 shows the I/O parameters when the technological parameters of the bearing are considered as equivalent.

### 2.4. Data Pre-Processing and Defining Datasets for Training, Validation and Testing

Prior to starting the work with neural networks, experimental data are often pre-processed which consists of linear scaling of the input data. Scaling the input parameters improves the training conditions of the networks in terms of achieving the optimal set of weight coefficients in the network. Linear scaling of the output parameters can establish a balance between outputs whose intensities are very different. Linear scaling of the output parameters was performed in the interval from 0.5 to 0.8 according to Equation (5). This scaled data were further divided into sets and used to train artificial neural networks.

(5) $$x_{skal}={\bar{x}}_{\min}+\frac{x-x_{\min}}{x_{\max}-x_{\min}}\cdot \left({\bar{x}}_{\max}-{\bar{x}}_{\min}\right)$$

**Figure 9:**
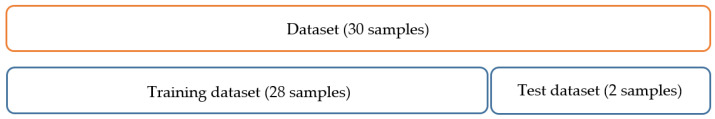
Data distribution.

The data were then divided into the training set, validation set and test set. In general, the most common data distribution ratio is 70% for the training set, 15% for the validation set, and 15% for the test set. These relations can be changed to obtain a better-quality model. In this paper, for the purpose of analyzing the dynamic behavior of bearings, sets were formed according to the number of bearings in a certain set. The total number of bearings was 30 with 2 sets allocated to post-training testing and network performance evaluation, as shown in Figure 9. The remaining 28 bearings were divided into 20 for training, 4 for validation and 4 for testing.

The evaluation of the model predictions is based on Pearson’s correlation coefficient (R), the determination coefficient (R^2^) and the average prediction error expressed as a percentage. The evaluation was calculated for each band separately. The overall evaluation was calculated as the cumulative value of the Pearson’s correlation coefficient and the separate coefficients of determination, and the prediction fault was calculated as the mean value for all three bands. If the model is absolutely correct, then the values of the coefficients will be 1 in each band, that is, their cumulative value will be 3, and the mean value of the prediction fault will be zero. The model whose cumulative values of the coefficients are closest to the maximum, and at the same time, has a minimum mean value of the prediction fault, is considered the best. When selecting the model to be used for the analysis of the dynamic behavior of the bearing, the priority in the evaluation is assigned to the networks that have the smallest average prediction fault, and as a secondary evaluation, the cumulative values of the coefficients are used.

### 2.5. Analyzed Models of Artificial Neural Networks

There are various architectures of neural networks from those with a single hidden layer with several neurons to networks with multiple hidden layers. The training was conducted with three different training algorithms: Levenberg–Marquardt (LM), Bayesian Regularization (BR), and Scaled Conjugate Gradient (SCG). Table 3 shows the analyzed artificial neural network models. The technological parameters of the bearing raceways were analyzed separately using equivalent values.

### 2.6. Method of Training Artificial Neural Networks

The training process of the artificial neural networks was carried out for each training configuration, which includes the training algorithm, the number of hidden layers, and the number of neurons in the hidden layer. A special training algorithm was created to enable automation of the training process.

### 2.7. Description of the Training Algorithm

**Figure 10:**
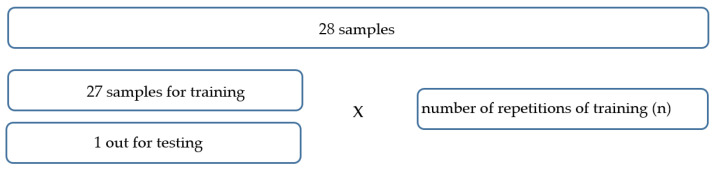
Schematic view of the training process.

One way to improve the performance of neural networks is to train a set of neural networks on the same data. This method is used to improve the generalization of artificial neural networks when noise is present and when a small amount of data is available. For this purpose, an algorithm was written to enable the training process to be repeated *n* times for a certain configuration (Figure 10). By executing the code, a set of neural networks is generated so that out of 28 bearings, the data for one are extracted for testing after training, and training is carried out with the remaining 27 bearings. The procedure is repeated until passing a cycle where all 28 bearings are separated. In this way, a set of 28 networks is obtained, and together they form one training cycle. The number of cycle repetitions can be set freely. In this paper, the maximum adopted number of repetitions is 10. Adopting more than 10 cycles requires more time for training networks, especially for multi-layered networks with 20 or more neurons in each layer. The training process was carried out according to the order listed in Table 3. Based on the number of repetitions, the results of training can range from one group of 28 networks to 10 groups of 28 networks. All of groups are tested and the best one is adopted.

**Figure 11:**
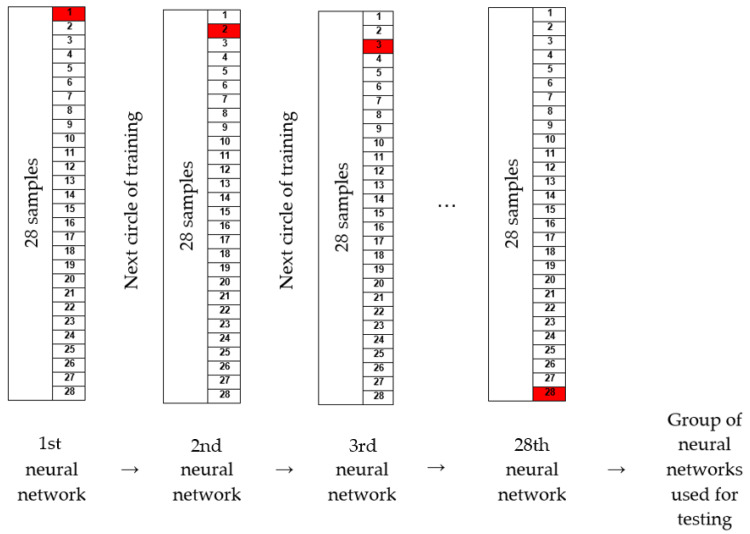
Process of training in a group and process of training algorithm (the red sample is out of training).

The process carried out in order to form a group of neural networks is shown schematically in Figure 11.

### 2.8. Selection of Artificial Neural Network Models

One set consists of 28 networks that are tested on two previously separated bearings. The adopted result is the mean value of the predicted vibration velocity amplitude in all three bands for all 28 networks. The separate average values of the prediction fault for all three bands, and the cumulative values of the Pearson’s correlation coefficient and the coefficient of determination are calculated for all 10 sets. Based on the calculated parameters, the sets are evaluated, and the set with the smallest fault and the highest values of Pearson’s correlation coefficient and coefficient of determination is adopted.

After calculating the parameters for evaluating the prediction quality of the artificial neural network, the model with the best prediction was selected. The model with the lowest prediction fault had one hidden layer with 11 neurons, and the scaled conjugate gradient (SCG) training algorithm was then applied. With this model, the technological parameters of the bearing were observed separately. The average prediction fault of the selected model was 8.1% in each frequency band. The adopted model was further used for the purpose of predicting the quality class of a new ball bearing and analyzing the influence of the surface roughness of the raceway and the internal radial clearance on the bearing vibrations.

The best performing model, where technological parameters were considered together (equivalent), had 3 hidden layers with 15 neurons in each layer, used a Levenberg–Marquardt training algorithm and achieved a prediction fault of 8.9%.

## 3. Results and Discussion

The results given in this section apply to a constant axial load of 200 N. The other parameters for bearing geometrics are taken from bearings in the training process.

### 3.1. Prediction of Quality Classes of Bearing

Table 4 presents the allowed vibration velocities of new ball bearing 6006 for low, medium and high-frequency bands. Class Q5 includes the best quality bearings, while Q7 corresponds to the lowest quality. It can be seen that there is large difference in vibration velocities between classes, and adopted models with prediction errors of less than 9% can fulfill the task and make an appropriate prediction.

### 3.2. Influence of the Surface Roughness of the Outer Ring

**Figure 12:**
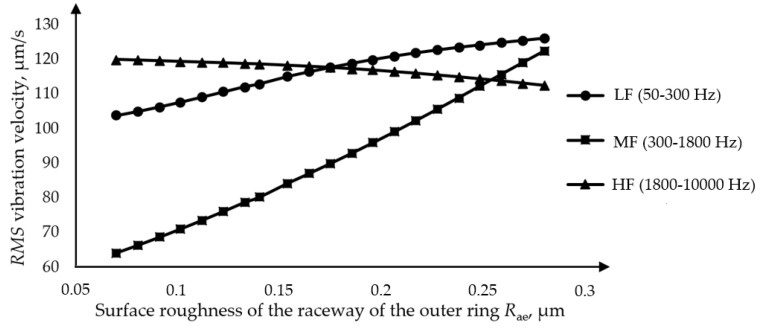
Dependence of the RMS value of the vibration velocity on the surface roughness of the raceway of the outer ring.

The analysis of the influence of the surface roughness of the outer ring was performed on one of two separate bearings, whereby the value of the surface roughness of the outer ring was adjusted at intervals from the minimum to the maximum value while the other parameters remained constant. Figure 12 shows the dependence of the RMS value of the vibration velocity for the three frequency bands on the amplitude of the surface roughness of the outer ring obtained by model 1. An increase in the amplitude of the surface roughness of the outer ring causes an increase in the RMS value of the vibration velocity in the low-frequency band, and the same trend is observed in the medium-frequency band. In the band of high frequencies, the opposite trend occurs, and there is a slight decrease in the amplitude of the vibration velocity. The obtained dependencies correspond to the experimentally obtained values. In the analysis model applied, the technological parameters of the bearing were observed separately.

Table 5 shows the RMS vibration velocities obtained by experimental measurement and the data obtained by the model predictions. For each value, the quality class is determined according to Table 4. The results are presented for bearings with different surface roughness values of the outer ring raceway. The comparison of measured and predicted class quality of the testing samples shows high model accuracy. In the low-frequency band, all the predictions are correct. In the medium- and high-frequency bands there are two misses, where the model predicted one class better quality because of the measured RMS vibration velocity was close to the boundary value. According to the rule of determination of bearing quality class, the worst class in the all the frequency bands must be adopted. For test sample 1, this is Q6, even though it reached Q5 in the medium-frequency band. For some predictions, there are significant differences between the predicted and measured values, but in these cases, the model still reached the correct prediction of bearing quality, which is the main aim of this research.

### 3.3. Influence of the Surface Roughness of the Inner Ring

**Figure 13:**
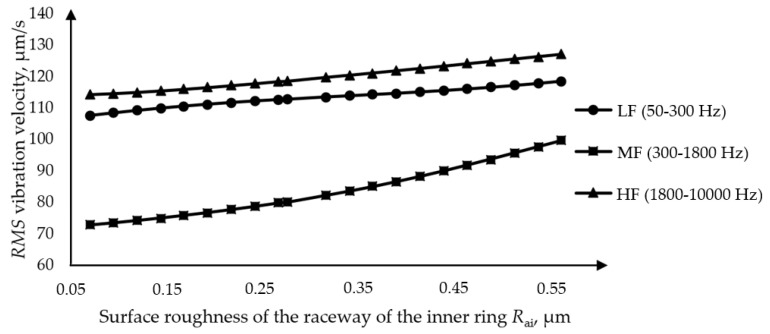
Dependence of the RMS value of the amplitude of the vibration velocity on the amplitude of the surface roughness of the raceway of the inner ring.

The analysis of the influence of the surface roughness of the inner ring was performed on one of two separate bearings, where the value of the surface roughness of the inner ring was adjusted at intervals from the minimum to the maximum value. The RMS values of the vibration velocity depending on the surface roughness of the inner ring raceway are shown in Figure 13. The increase in the amplitude of the surface roughness of the inner ring causes a slight increase in the amplitude of the vibration velocity in the bands of low and high frequencies. The amplitudes of the vibration velocity in the medium-frequency band have a slightly more significant growth with increasing surface roughness. In the analysis model applied, the technological parameters of the bearing were observed separately.

Table 6 shows the values of RMS vibration velocity obtained by experimental measurement and the values predicted by the model. For each value, the quality class is determined according to Table 4. The results are presented for bearings with different levels of surface roughness of the inner ring raceway. The comparison of measured and predicted class quality of the testing samples demonstrates the model’s high accuracy.

### 3.4. The Influence of Equivalent Surface Roughness and Radial Clearance

For the analysis of the influence of equivalent surface roughness and radial clearance in the bearing, in the applied model, the technological parameters of the bearing were considered as equivalent. The influence of equivalent surface roughness and radial clearance was analyzed on one of two separate bearings, where the value of the specified parameters was adjusted at intervals from the minimum to the maximum value while the other parameters remained constant.

**Figure 14:**
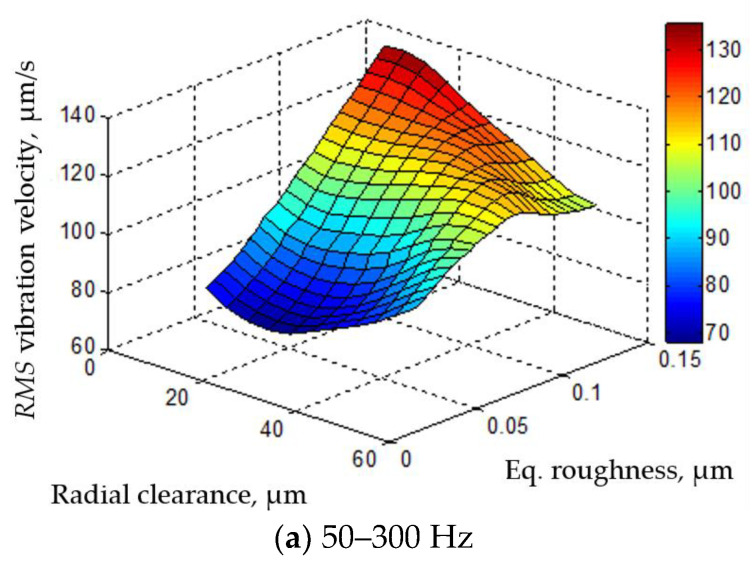

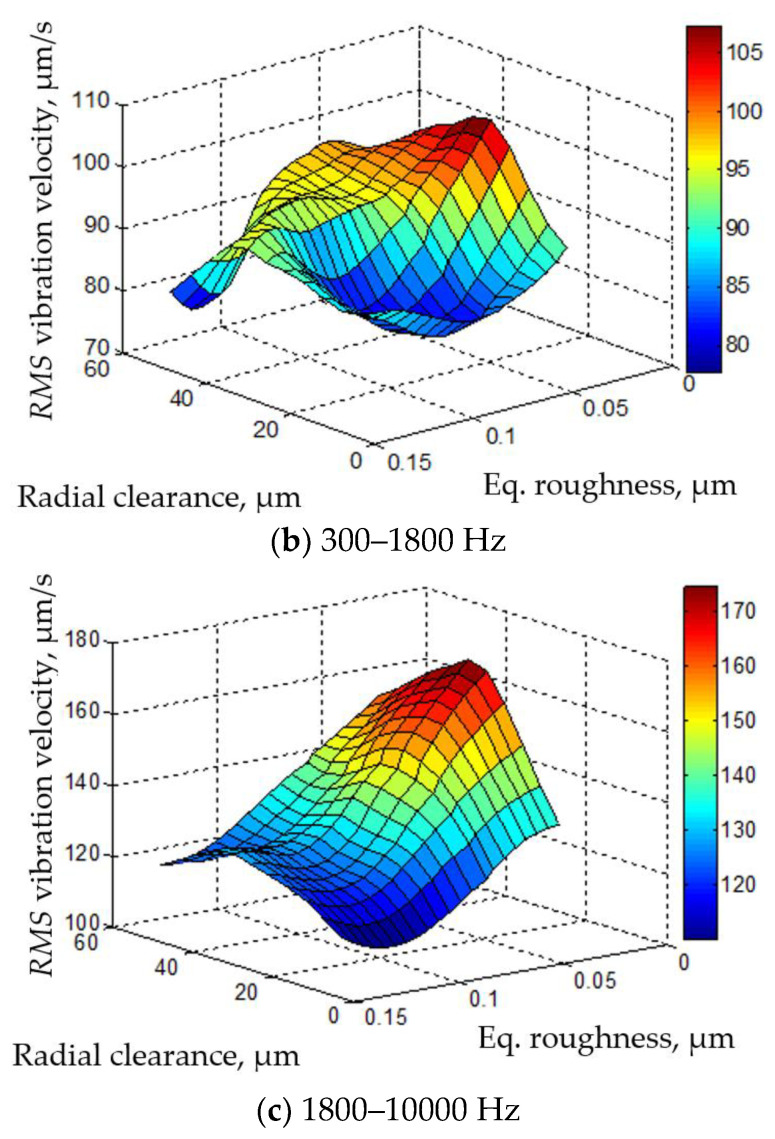
Mutual influence of radial clearance and equivalent roughness amplitude on RMS values of vibration velocity amplitude.

The mutual influence of the radial clearance and the equivalent roughness amplitude on the RMS value of the vibration velocity amplitude is shown in Figure 14. The minimum vibration velocity in the low- and medium-frequency bands was ensured when the radial clearance was approximately 20 μm. The minimum vibration velocity in all frequency bands was achieved at an equivalent roughness amplitude of 0.1 μm.

## 4. Conclusions

Based on the overall analysis of the results obtained using the neural network models, the following conclusions can be drawn:

- The adopted models are capable of predicting the quality class for new ball bearings and can reduce time required for quality control in bearing production;
- The increase in roughness on the outer raceway causes a significant increase in the vibration level in the medium-frequency band (300–1800 Hz) and a moderate increase in the low-frequency band (50–300 Hz), whereas the change in vibration level in the high-frequency band is negligibly small;
- An increase in surface roughness on the raceway of the inner ring has a negligible effect on the amplitude of the vibration velocity in the low-frequency band, and causes a moderate increase in the medium and high band. The growth in the newly introduced parameter of the equivalent roughness of the raceway affects the moderate growth in the amplitudes of the vibration velocity in the low-frequency band. In the medium-frequency band, the model predicts global minimum vibration velocities at an equivalent roughness amplitude of 0.1 µm. In the high-frequency band, there is a slight decrease in the velocity of vibrations with an increase in the amplitude of the equivalent roughness;
- The neural network model predicted that the minimum vibration level is obtained in all frequency bands if the radial clearance has amplitude of around 20 µm and the equivalent roughness has an amplitude of around 0.05 µm.

The research in this paper indicates the possibility of applying artificial neural networks for the analysis of the dynamic behavior of ball bearings, which has not been shown in the literature to date.

## Figures and Tables

**Table 1:** Main statistical characteristics of analyzed bearing parameters.

Bearing Characteristics	Minimum Value x_min_	Maximum Value x_max_	Mean Value $\bar{x}$	Standard Deviation *s*
Gr, [µm]	6	51	24.03	14.54
Ri/Re	0.95	0.98	0.96	0.01
Rae, [µm]	0.07	0.28	0.13	0.04
Rai, [µm]	0.07	0.56	0.26	0.1
Wte max, [µm]	0.1	1.97	0.8	0.32
Wti max, [µm]	0.25	0.66	0.39	0.13
Wte, [µm]	1.5	17.82	5.01	2.86
Wti, [µm]	0.4	2.63	0.92	0.41

**Table 2:** Main statistical characteristics of equivalent bearing parameters.

Bearing Characteristics	Minimum Value x_min_	Maximum Value x_max_	Mean Value $\bar{x}$	Standard Deviation *s*
R_a ekv_	0.04	0.14	0.08	0.02
W_t max ekv_	0.16	0.47	0.25	0.06
W_t ekv_	0.36	1.78	0.74	0.26

**Table 3:** Overview of analyzed models of artificial neural networks.

Analyzed Artificial Neural Network Models					
Technological Parameters Separately			Technological Parameters Equivalent		
Training algorithm			Training algorithm		
Levenberg– Marquardt	Bayesian Regularization	Scaled Conjugate Gradient	Levenberg– Marquardt	Bayesian Regularization	Scaled Conjugate Gradient
ANN architecture			ANN architecture		
One hidden layer (Number of neurons from 1 to 30)			One hidden layer (Number of neurons from 1 to 30)		
Two hidden layers (Number of neurons from 1 to 30)			Two hidden layers (Number of neurons from 1 to 30)		
Three hidden layers (Number of neurons from 1 to 30)			Three hidden layers (Number of neurons from 1 to 30)		

**Table 4:** Allowed vibration velocity for bearing 6006.

Bearing 6006	RMS of Vibration Velocity, µm/s Class Q7	RMS of Vibration Velocity, µm/s Class Q6	RMS of Vibration Velocity, µm/s Class Q5
Low-frequency band	224	112	71
Medium-frequency band	160	80	80
High-frequency band	450	224	112

**Table 5:** Experimental and predicted RMS values of vibration velocity for testing samples, measured quality class and predicted quality class based on surface roughness of the outer ring raceway.

Test Sample 1 Rae 0.09 µm			Test Sample 2 Rae 0.072 µm		Test Sample 3 Rae 0.125 µm		Test Sample 4 Rae 0.168 µm	
	Measured RMS of Vibration Velocity, µm/s, Class Quality	Predicted RMS of Vibration Velocity, µm/s, Class Quality	Measured RMS of Vibration Velocity, µm/s, Class Quality	Predicted RMS of Vibration Velocity, µm/s, Class Quality	Measured RMS of Vibration Velocity, µm/s, Class Quality	Predicted RMS of Vibration Velocity, µm/s, Class Quality	Measured RMS of Vibration Velocity, µm/s, Class Quality	Predicted RMS of Vibration Velocity, µm/s, Class Quality
Low-frequency band	80 Q6	105 Q6	60 Q6	103 Q6	73 Q6	108 Q6	97 Q6	83 Q6
Medium-frequency band	56 Q5	62 Q5	57 Q5	61 Q5	83 Q6	77 Q5	82 Q6	110 Q6
High-frequency band	121 Q6	119 Q6	107 Q5	118 Q6	115 Q6	118 Q6	132 Q6	117 Q6

**Table 6:** Experimental and predicted RMS values of vibration velocity for testing samples, measured quality class and predicted quality class based on the surface roughness of the inner ring raceway.

Test Sample 1 Rai 0.073 µm			Test Sample 2 Rai 0.157 µm		Test Sample 3 Rai 0.179 µm		Test Sample 4 Rai 0.271 µm	
	Measured RMS of Vibration Velocity, µm/s, Class Quality	Predicted RMS of Vibration Velocity, µm/s, Class Quality	Measured RMS of Vibration Velocity, µm/s, Class Quality	Predicted RMS of Vibration Velocity, µm/s, Class Quality	Measured RMS of Vibration Velocity, µm/s, Class Quality	Predicted RMS of Vibration Velocity, µm/s, Class Quality	Measured RMS of Vibration Velocity, µm/s, Class Quality	Predicted RMS of Vibration Velocity, µm/s, Class Quality
Low-frequency band	80 Q6	105 Q6	60 Q6	110 Q6	73 Q6	111 Q6	97 Q6	111 Q6
Medium-frequency band	56 Q5	71 Q5	57 Q5	71 Q5	83 Q6	78 Q5	82 Q6	84 Q6
High-frequency band	121 Q6	113 Q6	107 Q5	115 Q6	115 Q6	116 Q6	132 Q6	117 Q6

## Data Availability

The data that support the findings of this study are available from the corresponding author, upon reasonable request.
